# A descriptive summary of the WHO availability assessments of medical abortion medicines in eight African countries

**DOI:** 10.1186/s12978-024-01898-8

**Published:** 2024-12-27

**Authors:** Amy Grossman, Ndola Prata, Sarah Jones, Laurence Läser, Bela Ganatra, Antonella Lavelanet, Natalie Williams, Chilanga Asmani, Hayfa Elamin, Leopold Ouedraogo, Lucy Sejo Maribe, Dina Vladimirovna Gbenou, Yelmali Clotaire Hien, Moussa Dadjoari, Fousséni Dao, Mariette Claudia Adame Gbanzi, Robert Mulunda Kanke, Franck Biayi Kanumpepa, Dudu Dlamini, Grace Motsoanku Mefane, Sirak Hailu Bantiewalu, Mary Nana Ama Brantuo, Olive Sentumbwe-Mugisa, Richard Mugahi, Olumuyiwa Adesanya Ojo, Adeniyi Kolade Aderoba, Ulrika Rehnström Loi

**Affiliations:** 1 Venture Strategies for Health & Development/OASIS, Berkeley, CA USA; 2 Bixby Center for Population, Health & Sustainability, School of Public Health, University of California, Berkeley, CA USA; 3 UNDP‑UNFPA‑UNICEF‑WHO‑World Bank Special Programme of Research, Development and Research Training in Human Reproduction (HRP), Department of Sexual and Reproductive Health and Research, World Health Organization, 20 Avenue Appia, 1211 Geneva, Switzerland; 4 World Health Organization, Regional Office for Africa, Brazzaville, Republic of Congo; 5 World Health Organization, Botswana Country Office, Gaborone, Botswana; 6 World Health Organization, Burkina Faso Country Office, Ouagadougou, Burkina Faso; 7 Ministry of Health, Burkina Faso, Ouagadougou, Burkina Faso; 8 World Health Organization, Central African Republic Country Office, Bangui, Central African Republic; 9 Ministry of Health, Central African Republic, Bangui, Central African Republic; 10 World Health Organization, Democratic Republic of the Congo Country Office, Kinshasa, Democratic Republic of the Congo; 11 Ministère de la Santé Publique, Hygiène et Prévention, Democratic Republic of the Congo, Kinshasa, Democratic Republic of the Congo; 12 World Health Organization, Eswatini Country Office, Mbabane, Eswatini; 13 Ministry of Health, Lesotho, Maseru, Lesotho; 14 World Health Organization, Namibia Country Office, Windhoek, Namibia; 15 World Health Organization, Uganda Country Office, Kampala, Uganda; 16 Ministry of Health, Reproductive Health and Infant Health, Uganda, Kampala, Uganda

**Keywords:** Medical abortion, Mifepristone, Misoprostol, Combi-pack, Abortion, Botswana, Burkina Faso, Central African Republic, Democratic Republic of the Congo, Eswatini, Lesotho, Namibia, Uganda

## Abstract

**Background:**

The use of medical abortion using either a combination of mifepristone and misoprostol, or misoprostol alone has contributed to increased safety and decreased mortality and morbidity. The availability of quality medical abortion medicines is an essential component in the provision of quality abortion care. Understanding the factors that influence the availability of medical abortion medicines is important to help in-country policymakers, program planners, and providers improve availability and use of medical abortion.

**Methods:**

Using a national assessment protocol and an availability framework, we assessed the availability of medical abortion medicines across five elements (Registration & Quality Assurance, Policy & Financing, Procurement & Distribution, Provider Knowledge, and End-user Knowledge) in eight countries: Botswana, Burkina Faso, Central African Republic, Democratic Republic of the Congo, Eswatini, Lesotho, Namibia and Uganda between November 2020 and November 2021. The assessment included an online desk review and virtual or telephone-based key informant interviews.

**Results:**

Registration of medical abortion medicines—misoprostol or co-packaged mifepristone and misoprostol products (combi-pack)—was established in all countries, except the Central African Republic. In Lesotho and Eswatini, the national regulatory agency is still in development and importation of Cytotec™ misoprostol is permitted for off-label use in obstetrics/gynecology. Misoprostol was included in all countries’ essential medicines lists, except Botswana. Burkina Faso and Democratic Republic of the Congo also include mifepristone on their essential medicines list and medical abortion regimens in national abortion care service and delivery guidelines. Additionally, guidelines clarified health worker roles in the provision of abortion care specific to the legal context of each country and permitted task-shifting of abortion service provision. Where guidelines did not exist, medical abortion medicines and their use were not well integrated into the public health care system. Community awareness activities on abortion rights and services have been limited in scope across the countries assessed, however, end-users' awareness of misoprostol as a medical abortion medicine was reported.

**Conclusion:**

The national landscape assessments identified several cross-cutting opportunities to improve availability of medical abortion medicines, including importing quality-assured medical abortion medicines; developing nationally approved abortion service and delivery guidelines that optimize healthcare worker roles; and expanding communication strategies to reach end-users and pharmacists.

## Background

Recent estimates suggest that while the overall rate of unintended pregnancies has fallen, the proportion that ends in abortion has risen, largely driven by improved access to sexual and reproductive healthcare in high-income countries [1]. The combination of mifepristone followed by misoprostol, or misoprostol alone, are World Health Organization (WHO)-recommended methods for medical abortion (MA) that offer an alternative to surgical abortion [2]. Medical abortion has been shown to enhance autonomy, choice, and safety in abortion care. Medical abortion allows women greater control over the timing and setting of the procedure, leading to increased satisfaction and empowerment. The safety profile of medical abortion is also well-established, with low complication rates, making it a viable option for many women seeking abortion care [3, 4]. However, unsafe abortion—an abortion carried out by a person lacking necessary skills or in an environment that lacks minimal medical standards, or both—remains a public health challenge, particularly in low-income countries [5, 6]. Of the 55.7 million abortions that occurred globally each year between 2010 and 2014, about 45 percent were unsafe and nearly all took place in a developing country [6].

In recent years, the number of misoprostol-alone and co-packaged mifepristone and misoprostol (combi-pack) products that have been used for abortion has grown globally [7, 8]. Since 2014, five misoprostol and three mifepristone products and a combi-pack have been WHO prequalification (PQ)-listed, a global standard for quality assurance for pharmaceutical products [9]. The availability of quality-assured MA medicines (WHO PQ-listed products or those approved by a Stringent Regulatory Authority (SRA) [10]) is an essential component in the provision of quality abortion care. Understanding the factors that influence the availability of MA medicines is important to help policymakers, program planners, and providers in countries improve availability and use of quality medicines.

This paper builds upon the WHO’s landscape assessments on the availability of MA medicines, wherein “availability” means that a woman can request and receive a-quality-assured and affordable MA product or service when and where she needs it [11, 12]. This paper describes results from eight country assessments in Botswana, Burkina Faso, Central African Republic, Democratic Republic of the Congo, Eswatini, Lesotho, Namibia, and Uganda. The purpose of the landscape assessments was to generate evidence to support policy dialogue and policymaking that is specific to the needs of a given country, in order to improve the availability of MA medicines.

## Methods

Country selection criteria were based on discussions with WHO Regional Office for Africa and the UNDP-UNFPA-UNICEF-WHO-World Bank Special Programme of Research, Development and Research Training in Human Reproduction (HRP). Factors such as the burden of unsafe abortion and public health need, opportunities to increase access to MA medicines, and country requests were considered during the selection process. Botswana, Burkina Faso, Central African Republic, Democratic Republic of the Congo, Eswatini, Lesotho, Namibia, and Uganda were selected for the second round of national assessments. After a reading of each country’s abortion law, seven of eight countries were categorized as medium restriction countries; wherein abortion is permitted in cases of rape, incest, fetal impairment, and to preserve the general, physical, and/or mental health of the pregnant woman (Botswana, Burkina Faso, Eswatini, Lesotho and Namibia), in addition to economic or social reasons (Central African Republic and Democratic Republic of the Congo). Uganda permits abortion only in cases to save the woman’s life and as such, is characterized as highly restrictive.

We developed a country assessment protocol to guide the methodology of the national landscape assessments in 2019 [11]. The assessment protocol included the adaptation of an availability framework to MA medicines and abortion, a desk review, key informant interviews, and an analysis of the data to identify barriers and opportunities to improve MA availability. In the case of Lesotho and Namibia, approval or a waiver from an ethics committee was obtained. In the remaining countries the assessments were deemed not to constitute humans’ subjects research and ethical approval was not necessary. In all interviews, verbal informed consent to participate in assessment activities was obtained from all participants.

We organized the assessments and their findings around an availability framework, including Registration & Quality Assurance, Policy & Financing, Procurement & Distribution, Provider Knowledge, and End-user Knowledge, as described in detail previously [11]. Country assessments were conducted between November 2020 and November 2021. Data collection typically took between 12 and 14 weeks per country. Key informant interviews were held largely virtually or by telephone due to COVID-19 restrictions.

## Results

### Registration & quality assurance

**Fig. 1:**
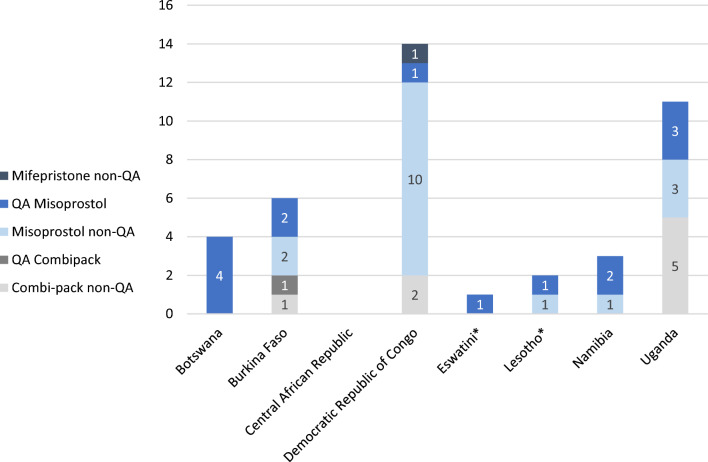
Number of registered MA medicines with and without quality assurance by country. *Health products are imported, not formally registered given the NRA is not fully operational

The national regulatory authority (NRA) in each of the eight countries approved the MA products shown in Fig. 1 While NRAs have their own quality standards, for the purpose of the assessments, a product with quality assurance is defined as one that is either WHO PQ-listed or approved by a SRA. Central African Republic had no MA medicines registered, whereas Democratic Republic of the Congo and Uganda had the greatest number of registered MA medicines with and without quality assurance, at 14 and 11 products, respectively (Fig. 1). Only one country, Burkina Faso, had a combi-pack (Medabon®, Sun Pharmaceuticals) that met the quality assurance criteria of the assessment. In Democratic Republic of the Congo and Uganda, the combi-packs that are currently registered are not WHO PQ-listed or SRA-approved but are approved for medical termination of pregnancy up to 63 days or amenorrhea; they are made by the Indian manufacturers, Naari, Synochem, and Acme Formulations. Democratic Republic of the Congo is the only country to have a registered mifepristone product (Prevent™ 10 mg and 50 mg/Synochem) in our sample, which is neither SRA-approved nor WHO PQ-listed, nor is it the correct dose used for MA.

We found that the use of WHO’s Collaborative Registration Procedure (CRP), which can enable accelerated regulatory approval in countries, was only used for MA medicines in Namibia. In Namibia, both misoprostol products, Celprotec™ and Avertiso™ (Acme Formulations), followed the WHO CRP pathway, and regulatory approval was granted within 90 days. In Lesotho and Eswatini the NRAs are not fully operational and the governments rely upon importation mechanisms of health products, not formal product registrations. Current importation requirements specify WHO PQ or SRA status of medicines, which will also be required for registration of new products when their NRAs are established. In both countries, Cytotec™ (Pfizer Pharmaceuticals) is imported, which is the originator misoprostol product and is not SRA-approved for MA, only the treatment of gastric ulcers. In Lesotho, a consignment of Cytomis™ misoprostol (Incepta Pharmaceuticals) was recently imported when the government could not secure Cytotec™ owing to global COVID-19 supply chain issues.

### Policy & financing

Standard treatment guidelines (STG) indicate rational and judicious use of medicines for specific health indications and national essential medicines lists (NEML) prioritize medicines to be procured for the public healthcare system [13]. National abortion care guidelines define who, when, where, and how abortion services are delivered in the country, and may be separate policy documents or included in STGs. The inclusion of the combination regimen in NEML, STGs, and abortion care guidelines varied across the countries assessed (Table 1). Mifepristone and misoprostol regimen for induced abortion is included on the NEML and abortion care service and delivery guidelines for Burkina Faso (2020) and Democratic Republic of the Congo (2020). Misoprostol regimens for prevention and treatment of postpartum hemorrhage and postabortion care (PAC) management are listed in the NEML and STG for Eswatini (2012), Lesotho (2017), and Namibia (2021), as well as the NEML of Uganda (2016). Botswana excludes misoprostol and mifepristone on its NEML; however, a Ministry-approved guideline and trainers’ manual on PAC (2013) includes the combination and misoprostol-alone regimens for induced abortion. In Uganda, national guidelines for abortion care and service delivery are outdated, and revised guidelines have been stayed by the Ministry of Health since 2015, owing to disagreements on the content and a need to consult further with stakeholders, including religious leaders [14, 15].

**Table 1:** Inclusion of MA medicines or protocols for their use in national policy documents

Country	MA medicines on NEML (Year)	Type of guideline that specifies MA protocols
Botswana	None (2012)	Comprehensive PAC Guideline & Trainer’s Manual, 2013
Burkina Faso	Mifepristone & Misoprostol (2020)	Reproductive Health Protocols, 2018
Central African Republic	Misoprostol (2017)^a^	None
Democratic Republic of the Congo	Mifepristone & Misoprostol (2020)	Standards and Guidelines for Comprehensive Female-Centered Abortion Care, 2020
Eswatini	Misoprostol (2012)^b^	None^c^
Lesotho	Misoprostol (2017)	None
Namibia	Misoprostol (2021)	None
Uganda	Misoprostol (2016)	National Policy Guidelines & Service Standards for Sexual & Reproductive Health & Rights, 2nd Ed, 2012

^a^In Central African Republic misoprostol is listed for gastric ulcers only, whereas misoprostol is included for postpartum hemorrhage and PAC on all other NEML when listed

^b^At the time of the assessment, the Ministry of Health was revising the STG/EML. The Sexual and Reproductive Health Interagency Technical Coordinating Committee is advocating for the inclusion of WHO-approved regimens (misoprostol- alone and the combination regimen) for MA and the inclusion of misoprostol on the EML for all obstetric indications, including TOP

^c^Draft Comprehensive Abortion Care Guidelines under development at the time of the assessment

### Procurement & distribution

MA medicines are not manufactured in any of the countries we assessed. We identified whether MA medicines are being procured for the public sector by a country’s central medical stores or imported by social marketing organizations (SMOs) and/or wholesale commercial distributors for the private sector. In all countries, misoprostol for postpartum hemorrhage and/or PAC had been procured at least once in the past three years for the public sector (Table 2). Only Democratic Republic of the Congo had ever procured a combi-pack product (Mifepak™) for its public health facilities.

**Table 2:** Importation of MA medicines for the public sector, private commercial sector and by social marketing organizations

Country	Misoprostol on NEML	Misoprostol in public sector	Misoprostol in private sector	Mifepristone on NEML	Combination regimen in public sector	Combination regimen in private sector
Botswana	–	Yes^a^	CD^a^	–	–	–
Burkina Faso	Yes	Yes	CD, SMO	Yes	–	CD, SMO
Central African Republic	Yes	Yes^b^	–	–	–	–
Democratic Republic of the Congo	Yes	Yes	CD, SMO	Yes	Yes	CD, SMO
Eswatini	Yes	Yes^a^	CD	–	–	–
Lesotho	Yes	Yes^a^	CD	–	–	–
Namibia	Yes	Yes	CD	–	–	–
Uganda	Yes	Yes	CD, SMO	–	–	CD, SMO

CD: commercial wholesale distributors; SMO: social marketing organization(s)

^a^Cytotec™ only

^b^CMS is bankrupt in Central African Republic and does not have a budget to procure essential medicines. UNFPA, Médecins Sans Frontières, Alliance for International Medical Action donate misoprostol to the public sector

Public sector distribution of MA medicines for induced abortion is limited to use by doctors at tertiary-level hospitals in all countries except Burkina Faso and Democratic Republic of the Congo. In both countries, national abortion care service delivery guidelines stipulate that trained providers at lower levels of the healthcare system can provide abortion services. Democratic Republic of the Congo also permits the use of MA at home according to the provider's monitoring instructions, if prescribed by a competent healthcare provider.

Distribution and use of misoprostol for treatment of incomplete abortion and miscarriage is permitted at lower levels of the healthcare system in Burkina Faso (Health and Social Promotion Centers and above), Central African Republic (NGO-use in humanitarian/ refugee settings), Democratic Republic of the Congo (Health Posts and above) and Uganda (Health Center 2 and above). In Botswana, Eswatini, Lesotho, and Namibia, distribution and use of misoprostol for PAC is restricted to hospitals by doctors. In these countries, misoprostol is often characterized as a “controlled drug, kept in a locked cupboard” with key informants citing concerns about potential pilfering and misuse.

Misoprostol is procured for the private sector in all countries assessed, except for Central African Republic. In Central African Republic, misoprostol is imported by NGOs and donated for public sector and humanitarian aid programs, not the commercial sector. The combi-pack is imported for the private sector in Burkina Faso and Democratic Republic of the Congo. Despite multiple combi-pack registrations in Uganda, including by SMOs for private sector use, distribution is limited owing to a restrictive policy environment and a lack of updated Ministry-approved safe abortion service delivery guidelines [14]. In Botswana, Eswatini, Lesotho, and Namibia, Cytotec™ is imported from neighboring South Africa for distribution in the private sector by a small number of commercial distributors; no SMOs socially market a product in these countries.

### Provider knowledge

Provider knowledge was assessed using proxies such as availability of ministry-approved training manuals and curricula and documented training efforts of healthcare workers [9]. Botswana, Burkina Faso, and Democratic Republic of the Congo have nationally approved training manuals that included MA regimens available [16–18]. In Namibia, at the time of the assessment, the Ministry of Health stated future plans to develop a training package that includes abortion care.

In Burkina Faso and Democratic Republic of the Congo, public–private partnerships have led to the development of a comprehensive abortion care roadmap and implementation and training plan specific to the public health context and workforce of each country. SMOs also have conducted several in-service trainings for private sector providers and pharmacists. In Botswana, Central African Republic, Eswatini, Lesotho, and Namibia, no government-supported training on comprehensive abortion care for public sector providers nor NGO-led in-service trainings for private sector providers, had occurred at the time of the assessment. According to one key informant in Lesotho, “*abortion would need to be legalized before national or district level training of providers would be possible.*” However, in each of these countries, abortion is legally permitted in cases of rape, incest, fetal anomaly, and to save a woman’s life or preserve her general health.

In Botswana, the *Comprehensive PAC Trainer’s Manual* (2013) includes mention of MA regimens but annual ministry-led trainings on PAC for public sector doctors, midwives, and nurses focus on misoprostol and MVA for treatment of incomplete abortion and post-abortion family planning, not induced abortion [16]. Moreover, neither mifepristone, nor combi-pack is available in the country. The ministries of health of Eswatini, Lesotho, and Namibia have trained a limited number of providers, mostly doctors and midwives at hospitals, on misoprostol and MVA as part of public–private supported PAC trainings. PAC is already included as part of pre-service training for nurses in Lesotho and in Burkina Faso, MVA and misoprostol protocols for treatment of incomplete abortion is taught at the National School of Public Health and to health attachés in obstetric and gynecological care.

In Uganda, the government focuses its training efforts on PAC, despite previously validated national abortion care guidelines that define the legal grounds and medical conditions for termination of pregnancy to save a woman’s life. Key informant interviews suggest that there has never been a national level training for public sector providers on comprehensive abortion care and there are currently no trainings planned, owing to the restrictive policy environment and continued disagreement on the content of revised national abortion care guidelines. According to the Ministry, “*With regard to in-service training, PAC and post abortion family planning refreshers are undertaken all the time. The only one that is not done is termination of pregnancy or MA due to the legal barriers and criminalization of abortion.*” In Uganda, as in Democratic Republic of the Congo and Burkina Faso, NGOs are taking the lead on training on MA and MVA for abortion care within the legal framework, with the majority financed and facilitated by SMOs in the private sector.

### End-user knowledge

In the countries assessed there is limited data on women’s knowledge of their country’s respective abortion laws or services. In Botswana, a 2019 study found that most respondents had limited or no knowledge of the country’s abortion law, irrespective of background and location. Where there was no knowledge, the default was to assume that abortion was illegal [19]. This was the general perspective of key informants working in government, NGOs, pharmacies, and service provision in the other countries as well. Key informants generally believed that the level of information available to women on abortion is very low and the majority are unaware that abortion is possible and permitted by law to save a woman’s life, preserve general health or in cases of rape and incest (in all countries except Uganda). Key informants across all countries reported that abortion stigma was common and driving the practice towards less safe methods or to self-induce with medications in the absence of knowledge of how to self-manage or of quality products and how to use them.

Key informants report increasing awareness among abortion-seekers of MA medicines, specifically misoprostol, and that awareness is generally higher in the capital cities. They suggested that women living in more urban settings with access to technology are more informed about MA. A 2020 unpublished study in Democratic Republic of the Congo found that the majority of girls and women interviewed in Kinshasa knew of at least one method of abortion, with misoprostol/Cytotec™ being the best known [20].

*“The combi-pack is an extremely new drug on the market and you can only find it in very few pharmacies, not in drug-shops, not in clinics, not in public health facilities. Very few women and even health workers know about it and how to use it well. It is expensive, so many prefer to use the more known misoprostol that is more available and cheaper. Most girls and young women get information about these pills from Google, peers and referrals. They access these commodities without much knowledge about them.”*

- Key Informant Interview, Uganda.

*“Women and adolescent girls in cities are increasingly aware and know mostly misoprostol as a MA product because they have more channels of information through social media platforms disseminated by human rights associations and NGOs.”*

-Key Informant Interview, Burkina Faso.

*“The vast majority of the cases we see in the hospital are from backstreet abortions… they come in having had an intervention. For example, having used misoprostol they bought on the black market – it can be found anywhere.”*

-Key Informant Interview, Botswana.

Women’s awareness of misoprostol was corroborated by a limited number of pharmacy visits in each country. Every pharmacy visited in Burkina Faso (n = 2), Democratic Republic of the Congo (n = 6), Lesotho (n = 2), Namibia (n = 2), and Uganda (n = 4) reports multiple clients requesting misoprostol monthly. In Botswana, three of the five pharmacies visited declined to share any information on MA products, while one pharmacy stated they did not carry misoprostol because there is “too much conflict and not enough demand”; the fifth shared that one to two clients a month request Cytotec™ specifically. In Eswatini, five of six pharmacies sampled, report up to 10–12 women a month requesting misoprostol, but only one pharmacy carries misoprostol, which was out of stock at the time of the assessment. MA medicines are unavailable in the private sector in Central African Republic. In Botswana, Eswatini, and Namibia, fear of legal retribution for dispensing misoprostol is cited by pharmacists surveyed, which prompted some pharmacies to not stock misoprostol or to create additional barriers to availability.

*“Women must have a prescription for misoprostol, and we usually require the doctor to call ahead in advance to say they are sending a patient ahead to us, rather than a patient walking in with a prescription with no advanced warning. If someone came in requesting misoprostol or had a prescription but no way of contacting the doctor who prescribed it, we would tell them that misoprostol is not available.”*

-Private pharmacist, Botswana.

Community awareness activities on abortion rights and services, including MA, have been limited in scope across the countries assessed or non-existent. Generally, across all countries, governments focus information, education, and communication efforts on comprehensive sexuality education aimed at reducing unintended pregnancy, omitting information on abortion rights and services specifically, as abortion is considered “taboo” and “fraught with stigmatization.”

*“There is absolutely zero community engagement. None whatsoever; as the perception is that it would be deemed as though [the Ministry] would be promoting abortion.”*

- Key Informant Interview, Botswana.

Small-scale efforts to sensitize communities on abortion rights and services via online campaigns, telemedicine and mobile apps, helplines, and/or community health workers are being utilized in Burkina Faso, Democratic Republic of the Congo, Namibia, and Uganda. Key informants also cite informal networks, the internet, pharmacies, and word of mouth as sources for women’s knowledge and access to abortion, including MA.

## Discussion

This paper describes the results of eight country assessments aimed at understanding the factors that influence the availability of MA medicines. Our assessment reinforced findings from previous country assessments that showed the registration of combi-pack for MA is possible irrespective of the legal framework for abortion in restrictive countries in Africa and Latin America [12, 21, 22]. Uganda, a country with a highly restrictive abortion law, had the second highest number of registered MA medicines, including five combi-packs and six misoprostol products. The role of civil society organizations in accelerating the introduction and roll-out of misoprostol for postpartum hemorrhage in Uganda has been documented elsewhere [22] and lends credence to the role of market shaping efforts even in highly restrictive contexts to register multiple MA medicines, including the combi-pack for abortion. An argument can be made that MA products should be widely available irrespective of legal frameworks. Good quality MA products can contribute to making abortion safer.

In our assessments, where SMOs are present, there is a combi-pack registered. Whereas misoprostol is imported by commercial wholesale distributors in the absence of an SMO presence in Botswana, Eswatini, Lesotho, and Namibia. However, misoprostol is not well integrated into the healthcare system, and distribution is limited to tertiary facilities in these countries and a limited number of pharmacies. Moreover, Cytotec™, the innovator misoprostol product approved for gastric ulcers only, was the dominant product imported. Off-label use of Cytotec™ is common in obstetrics and gynecology, but “off-label” means product inserts do not include dosage and administrative route information for unapproved clinical indications such as treatment of incomplete abortion and miscarriage, postpartum hemorrhage management and for induced abortion [23]. Providers must rely on clinical training, STGs, or national abortion care guidelines for treatment protocols using such medicines, which were mixed across countries.

The assessment identified the opportunity to support in-country registration of additional mifepristone and misoprostol products that are approved by an SRA or are WHO PQ-listed, using the Collaborative Registration Procedure or other regional regulatory reliance mechanisms to increase the availability of quality-assured products. Encouragingly, China Resources Zizhu Pharmaceuticals has entered into an agreement with DKT WomanCare to facilitate the registration of its WHO PQ-listed combi-pack product globally [24]. The Collaborative Registration Procedure agreement has only been signed by a few national regulatory agencies in our sample. It is envisioned that with greater participation in CRP, duplicative regulatory processes can be eliminated and registrations of quality-assured products can be accelerated. WHO is providing support and training to regulators to streamline the process.

We found that where national abortion care guidelines were approved by the Ministry, mid-level providers at lower levels of the healthcare system were trained to provide abortion services, as in Burkina Faso and Democratic Republic of the Congo. In Botswana, Eswatini, Lesotho, and Namibia, use of misoprostol for PAC is restricted to doctors, despite global guidance for optimizing health worker roles to improve access to life-saving emergency obstetric care [2]. These countries—Botswana, Eswatini, Lesotho, and Namibia—lacked national comprehensive abortion care guidelines. Healthcare worker knowledge about abortion legislation and clinical practice guidelines influence the provision of quality of care [25–29]. In Uganda, the Ministry’s stay on revised national guidelines has created confusion among service providers on what is permissible by law [14, 15].

In our assessments, the perception of key informants is that women are seeking MA medicines, commonly misoprostol, in the private sector for self-administration, as reported in other countries [20, 30–32]. Pharmacy workers interviewed corroborated that women regularly request misoprostol. Democratic Republic of the Congo was the only country in our sample that made formal allowances for the role of self-administration of MA medicines under the supervision of a healthcare provider [18]. Given its safety and ease of use, WHO includes self-administration with MA up to 12 weeks in its recommendations [33]. Effective use of MA medicines requires that the user receive accurate information, counseling, and quality medicines [2, 33, 34]. Internet sites and hotlines may be filling an information void on how to access and use these medications [34, 35]. However, our assessments also revealed reticence on the part of some pharmacists to stock MA medicines out of fear of legal retribution for dispensing misoprostol or stigma associated with the products, as documented elsewhere [36]. Abortion stigma impacts access to and the provision of quality, safe abortion care [37–39]. Fear of litigation and deeply entrenched abortion-related stigma influenced providers’ willingness to offer services and commercial distributors’ interest to stock or promote MA products in our sample. The assessments identified the need for more direct-to-consumer community awareness campaigns to inform end-users about their rights within the legal framework, the availability of safe abortion services or accurate mobile-based technologies for information and counseling on MA.

### Strengths and limitations

As previously reported, a strength of the assessments is that the holistic approach is useful to governments and program partners who may only be active in one component area of availability [11]. Importantly, we assessed both the commodity supply-side components with provider and end-user knowledge, including a limited number of pharmacy visits. While pharmacy visits were mostly in the capital cities and not representative, they did bolster key informants’ perspectives that there is awareness of misoprostol in the urban centers. Future assessments would be bolstered by secret shopper visits or formal inventory assessments at a representative sample of urban, peri-urban, and rural retail outlets and public sector pharmacies. We limited our inquiry to those products formally registered by the NRA of each country. Assessing the availability of unregistered products, pricing, and prescription-status was outside the scope of the assessments but is documented elsewhere [8]. The national landscape assessments present some additional limitations. The COVID-19 pandemic required governments to apply measures such as lockdowns and travel restrictions, this allowed for only a limited number of face-to-face interviews and most interviews took place virtually. Key informants were not always willing and/or available to discuss what they deemed a sensitive topic like MA, especially by email survey or phone. We acknowledge potential participation bias in those willing to speak on the topic, however we sought to mitigate this by seeking Ministry-approval and support. End-user knowledge was assessed by proxies relying on the available published literature and key informant interviews, instead of formal knowledge, attitudes and practices surveys, which were outside of the scope of the assessments. The assessments revealed a paucity of data on country-level abortion statistics and abortion-seekers knowledge and practices in each country. This information gap is cited elsewhere and an area in need of inquiry [7, 12]. We recognize that end-user knowledge relies upon other important potential barriers such as knowledge of where to access services and medicines, transportation, distance to and cost of services and medicines [34, 39].

Despite some limitations, several cross-cutting opportunities that are impacting the availability and use of MA in each country context were identified. These included the approval and import of quality-assured MA medicines, including combi-pack; the development and dissemination of nationally-approved comprehensive abortion care service and delivery guidelines that operationalize the abortion law and optimize healthcare worker roles, including mid-level providers; and recognizing the role of self-managed abortion by expanding information and communication models that reach women and providers alike, including pharmacists who are often first-line points of contact for abortion-seekers [2].

## Conclusion

The availability of quality-assured medicines is an essential component of abortion care. In order to improve availability, the assessments show the need to work across all five components of the availability framework—registration and quality assurance, policy and financing, procurement and distribution, provider knowledge, and end-user knowledge—while defining MA medicines’ use for abortion care specifically. The national landscape assessments can serve as a resource for countries to develop actionable strategies to ensure the availability of medical abortion medicines, including importing quality-assured medical abortion medicines; developing nationally approved abortion service and delivery guidelines that optimize healthcare worker roles; and expanding communication strategies to reach end-users and pharmacists with accurate information on the laws governing abortion, access to care, and products and their use.

### About this supplement

This article has been published as part of Reproductive Health Volume 20 Supplement 1, 2023: Availability of quality-assured medical abortion medicines. The full contents of the supplement are available online at https://reproductive-health-journal.biomedcentral.com/articles/supplements/volume-20-supplement-1.

## Data Availability

The data that support the findings of this study are available from the corresponding author, LL, upon reasonable request.

## Abbreviations

MA: Medical abortion
NEML: National essential medicines list
NRA: National regulatory authority
PAC: Postabortion care
SMO: Social marketing organizations
SRA: Stringent regulatory authority
STG: Standard treatment guidelines
WHO PQ: WHO Prequalification

## Declarations

### Disclaimer

The views expressed in this article are those of the authors and do not necessarily represent the views of, and should not be attributed to, the World Health Organization. The mention of specific companies or of certain manufacturers’ products does not imply that they are endorsed or recommended by WHO in preference to others of a similar nature that are not mentioned. Errors and omissions excepted the names of proprietary products are distinguished by initial capital letters.
